# Supplementary material for: Lifestyle parameters of Japanese agricultural and non-agricultural workers aged 60 years or older and less than 60 years: A cross-sectional observational study
Source: PLoS One. 2023 Oct 4;18(10):e0290662. doi: 10.1371/journal.pone.0290662 (PMC10550184; doi:10.1371/journal.pone.0290662)
Supplement: S1 Appendix — (DOCX) [file pone.0290662.s001.docx]

**S1 Appendix**

**Table A. Proportion of participants engaged in each occupation**

|  | Age <60 years | Age ≥60 years |
| --- | --- | --- |
|  | n=560 | n=251 |
|  | n (%) | n (%) |
| Agricultural job | 135 (24.1) | 140 (55.8) |
| Professional and technical job | 63 (11.3) | 21 (8.4) |
| Managerial job | 26 (4.6) | 8 (3.2) |
| Clerical job | 102 (18.2) | 10 (4.0) |
| Sales job | 53 (9.5) | 15 (6.0) |
| Service job | 80 (14.3) | 36 (14.3) |
| Security job | 6 (1.1) | 2 (0.8) |
| Forestry job | 2 (0.4) | 0 (0) |
| Fishery job | 1 (0.2) | 0 (0) |
| Transportation and communications job | 9 (1.6) | 5 (2.0) |
| Manufacturing job | 88 (15.7) | 16 (6.4) |

*Seven cases with multiple selections were counted as both.
