# Supplementary material for: Lifestyle parameters of Japanese agricultural and non-agricultural workers aged 60 years or older and less than 60 years: A cross-sectional observational study
Source: PLoS One. 2023 Oct 4;18(10):e0290662. doi: 10.1371/journal.pone.0290662 (PMC10550184; doi:10.1371/journal.pone.0290662)
Supplement: S2 Appendix — (DOCX) [file pone.0290662.s002.docx]

**S2 Appendix. Details of measurements**

***Questionnaire***

Data on working days per week, working hours per day, regular physical activity and exercise habits, smoking status (current, former, no), drinking habits (current, former, no), bedtime, wake-up time, sleeping hours, napping habit (yes or no), number of family members living together, presence of spouse (yes or no), number of people who are socially connected, proportion of time spent in each daily activity, regular dental checkups (yes or no), and frequency of toothpaste use were collected through a questionnaire.

Working days per week were categorized as more than 5 days or not.

Working hours per day were categorized as more than 8 h or not because the Labor Standards Law in Japan stipulates that statutory working hours are 8 h a day, 40 h a week.

Proportion of time spent in each daily activity encompassed 18 activity options: “Personal chores,” “Eating,” “Commuting to work or school,” “Travel,” “Schoolwork,” “Housework,” “Caring or nursing,” “Childcare,” “Shopping,” “Watching TV/listening to the radio/reading newspapers or magazines,” “Rest and relaxation,” “Learning/self-education/training,” “Hobbies and amusements,” “Sports,” “Volunteer activities,” “Socializing,” “Medical examination and treatment,” and “Other.” Time spent on “Schoolwork” was not analyzed because this study was designed for occupational workers.

Daily activity was categorized as housework and related work (housework, caring or nursing, child care, and shopping), passive leisure activities (watching TV/listening to the radio/reading newspapers or magazines, rest and relaxation), and active leisure activities (learning/self-education/training, sports, hobbies and amusements, and volunteer activities). Moreover, participation in learning/self-education/training, sports and hobbies, and amusements was defined by whether the proportion of time spent in each daily activity was 0 or not. Participation in volunteer activities was not analyzed because the number of participants was small.

***Measurement of health-related quality of life***

The score for each subscale was calculated as 100 × (score − lowest score that the score can take) / (score range that the score can take) and was standardized by considering the Japanese population, with a mean of 50 and standard deviation of 10. Physical functioning; role limitations attributable to physical problems (RP), bodily pain (BP), and general health; and mental subscales, including vitality (VT), social functioning (SF), role limitations attributable to emotional problems (RE), and mental health were included in the questionnaire. The physical and mental component summary scores (PCS and MCS) were then calculated.

***Measurement of Pittsburgh Sleep Quality Index (PSQI)***

PSQI is a self-reported assessment of subjective sleep quality over the past 4 weeks and consists of 18 questions, which include seven sleep components (quality, latency, duration, efficiency, disturbance, medication, and daytime dysfunction); the scores range from 0 to 3, which are summed to yield a global score. Sleep disorder was defined as a PSQI global score ≥6.

***Measurement of nutritional status***

The brief (self-administered) diet history questionnaire (BDHQ) estimates dietary intakes of 58 food and beverage items in the preceding month. We assessed total energy intake; percentage of carbohydrates, proteins, and lipids; and salt intake.
