# Supplementary material for: Lifestyle parameters of Japanese agricultural and non-agricultural workers aged 60 years or older and less than 60 years: A cross-sectional observational study
Source: PLoS One. 2023 Oct 4;18(10):e0290662. doi: 10.1371/journal.pone.0290662 (PMC10550184; doi:10.1371/journal.pone.0290662)
Supplement: Attachment — Submitted filename: Response_to_Reviewers_230804.docx [file pone.0290662.s004.docx]

**Response to Editor’s comments**

Thank you for submitting your manuscript to PLOS ONE. After careful consideration, we feel that it has merit but does not fully meet PLOS ONE’s publication criteria as it currently stands. Therefore, we invite you to submit a revised version of the manuscript that addresses the points raised during the review process.

Thank you for the comment and invitation to revise the manuscript once more. Our point-by-point responses to the reviewer comments for this round, for which we would like to show our deepest appreciation, are given below.

**Response to Reviewer #2’s comments**

Unclear points which I pointed out were mostly addressed. I have no further comment for this manuscript.

Thank you very much for your important comments and re-consideration of our paper for publication.

**Response to Reviewer #3’s comments**

I have enjoyed reading your manuscript. here are few suggestions to make it better.

We would like to humbly oblige your additional suggestions, as they were valuable and helped enhance the quality of our paper. We have responded to your points below.

Major point

1) how did you assess "time spent on each daily activity". Please clarify this in the method section, then add a unit the proper unit of measurement within the tables where you report these results.

*(Reply)*

We thank you very much for this comment. For the proportion of time spent on each daily activity, the number of days and hours per week that the participant spent performing each activity were collected through a questionnaire, and the percentage of time spent on each daily activity per week was calculated. We have added this explanation to the manuscript (Method, Measurement subsection, lines 113–116) and in the S2 Appendix, and have added the unit pertaining to this variable (%) to Table 2.

*(Revisions)*

Pages 6–7, lines 113–116 of the revised manuscript:

For the proportion of time spent on each daily activity, the number of days and hours per week that the participant spent performing each activity were collected through a questionnaire, and the percentage of time spent on each daily activity per week was calculated. Data were collected in 18 categories as described in the S2 Appendix.

Page 1 of the revised S2 Appendix:

Proportion of time spent in each daily activity encompassed 18 activity options: “Personal chores,” “Eating,” “Commuting to work or school,” “Travel,” “Schoolwork,” “Housework,” “Caring or nursing,” “Childcare,” “Shopping,” “Watching TV/listening to the radio/reading newspapers or magazines,” “Rest and relaxation,” “Learning/self-education/training,” “Hobbies and amusements,” “Sports,” “Volunteer activities,” “Socializing,” “Medical examination and treatment,” and “Other.” Time spent on “Schoolwork” was not analyzed because this study was designed for occupational workers.

2) I suggest combining table 2 & 3 to be similar to table 4 for better prestation of the data

*(Reply)*

Following your suggestion, Tables 2 and 3 were combined into a single Table 2 in the revised manuscript.
